# Risk Factors of Developing Postinfectious Irritable Bowel Syndrome in Shigellosis Patients, 5 Years After Hospitalization During the Outbreak

**DOI:** 10.1093/ofid/ofae032

**Published:** 2024-01-22

**Authors:** Maryam Soheilipour, Atefeh Chahichi, Hamid Mohajer, Niousha Ghomashi, Hamidreza Roohafza, Peyman Adibi

**Affiliations:** Isfahan Gastroenterology and Hepatology Research Center, Isfahan University of Medical Sciences, Isfahan, Iran; Isfahan Gastroenterology and Hepatology Research Center, Isfahan University of Medical Sciences, Isfahan, Iran; Faculty of Medical School, Najafabad Branch, Islamic Azad University, Isfahan, Iran; School of Medicine, Isfahan University of Medical Sciences, Isfahan, Iran; Isfahan Cardiovascular Research Center, Isfahan University of Medical Sciences, Isfahan, Iran; Isfahan Gastroenterology and Hepatology Research Center, Isfahan University of Medical Sciences, Isfahan, Iran

**Keywords:** dysentery, gastroenteritis, postinfectious irritable bowel syndrome, *Shigella*

## Abstract

**Background:**

Irritable bowel syndrome (IBS) can be triggered by bacterial dysentery. This study aimed to investigate postinfectious IBS and its risk factors after the shigellosis outbreak in hospitalized patients.

**Methods:**

This retrospective study was conducted in 2020–2021 in referral hospitals for *Shigella* gastroenteritis during the 2014 shigellosis outbreak in Isfahan. The *Shigella*-infected group included hospitalized shigellosis patients with clinical symptoms and positive stool culture. The control group included patients matched pairwise on age and sex to the *Shigella*-infected group, admitted to the same hospitals in the same period with diagnoses other than shigellosis. Both groups had no history of diagnosed IBS before the outbreak. The incidence of IBS (according to Rome-III criteria) and its related factors was compared between the 2 groups 5 years after infection.

**Results:**

Of 619 participants, 220 (35.5%) were in the *Shigella*-infected group. The 5-year incidence of IBS was 31.8% and 5.7% in the *Shigella*-infected and control groups, respectively. Multivariate analysis showed that shigellosis was significantly associated with increased risk of IBS (odds ratio [OR], 17.18 [95% confidence interval {CI}, 9.37–31.48]). Multivariate analysis indicated education level (OR, 4.15 [95% CI, 1.47–11.73]), diarrhea lasting >4 days (OR, 1.69 [95% CI, 1.17–2.44]), and abdominal cramps during the infection (OR, 0.27 [95% CI, .77–.95]) associated with postinfectious IBS (*P* < .05).

**Conclusions:**

Hospitalized patients with *Shigella* gastroenteritis are at increased risk of IBS within 5 years after infection. Factors such as higher education level and the absence of abdominal cramps and diarrhea persisting for >5 days during hospitalization can further increase this risk.

## Teaser

Hospitalization due to *Shigella* gastroenteritis can strikingly increase the 5-year risk of developing irritable bowel syndrome. Clinical and background factors, such as education level, abdominal cramps, and prolonged diarrhea during hospitalization, can affect this risk.

Irritable bowel syndrome (IBS) is a common chronic digestive disorder with abdominal pain and changed bowel habits [1]. Rome-IV is the most recent criterion used for diagnosing IBS [2]. IBS can significantly impact patients’ lives and the healthcare system [3]. The prevalence of IBS is approximately 3.8% globally, 9.5% in Asia, and 17.5% in Latin America [4, 5]. IBS, which is more common among women and youth [6, 7], has several risk factors, such as intestinal infection and inflammation, genetics, changes in the intestinal microbiota, diet, anxiety, depression, bile acid metabolism, and impaired carbohydrate absorption [8, 9]. Some patients develop IBS following intestinal infections with bacterial, viral, or parasitic pathogens [10, 11]. About 10% of patients report the onset of IBS after an episode of dysentery [12]. Infectious gastroenteritis can cause microbial imbalance, mucosal layer dysfunction, sensitization of neurons, and activation of the immune system [13, 14]. Postinfectious IBS (PI-IBS) refers to the sudden appearance of new IBS symptoms following an episode of acute infectious gastroenteritis [12]. The incidence of PI-IBS depends on factors such as the involved pathogens, the geographic area, and the criteria used for IBS diagnosis [15]. In different studies, the prevalence or incidence of this disease has been estimated to be 5%–32% [16]. Previous studies have identified factors such as female sex, younger age, antibiotic therapy, diarrhea for >7 days, abdominal pain, and vomiting during PI-IBS infection. However, these results have been conflicting [16–19]. Although viral and protozoal agents can be involved in the pathogenesis of PI-IBS, most cases of PI-IBS develop after bacterial infections with *Campylobacter jejuni*, *Shigella sonnei*, *Salmonella enterica*, *Clostridioides difficile*, and *Escherichia coli* [17–19].

Although acute gastroenteritis is common in several developing Asian countries [20], data on PI-IBS from these regions are scarce. According to a 10-year follow-up study in South Korea, shigellosis patients had an increased risk of developing IBS in 1 and 3 years following the infection compared to controls [21].

In 2014, an outbreak of shigellosis occurred in Isfahan City. *Shigella* is one of the leading causes of bloody diarrhea among all ages worldwide and is responsible for 13% of all diarrhea-related deaths [22]. This retrospective cohort study aimed to assess the incidence of PI-IBS and identify its risk factors, 5 years after a shigellosis outbreak in Isfahan province in 2014, due to the limited research on PI-IBS following *Shigella* infection.

## METHODS

### Study Design and Patients

This retrospective cohort study was conducted in 2 referral hospitals for *Shigella* gastroenteritis in Isfahan (Shariati and Gharazi hospitals) in 2020–2021. For the *Shigella*-infected group, inpatients aged >16 years with clinical symptoms of shigellosis (fever, bloody diarrhea, tenesmus, and severe abdominal pain) and positive stool culture were enrolled. The control group included inpatients in the same hospitals during the outbreak with a diagnosis other than gastroenteritis, matched pairwise with the *Shigella*-infected group regarding age and sex. Both groups had no history of diagnosed IBS or other intestinal diseases, such as inflammatory bowel disease and celiac disease, before the outbreak. Patients with coinfection with other organisms responsible for IBS (eg, *Campylobacter* spp, *E coli*, and *C difficile*) through stool culture tests, incomplete medical records, past medical history of IBS before the outbreak, or medical history of inflammatory bowel disease or malignancies were excluded from the study. During hospitalization, patients received appropriate treatment at the discretion of the gastroenterologist and recommended treatments. Specifically, they were administered intravenous ceftriaxone, in line with the accessible antibiotics at the time.

### Patient Consent Statement

This study was designed and performed in accordance with the ethical standards as presented in the 1964 Declaration of Helsinki and its later amendments. The design of this study was approved by the Isfahan University of Medical Sciences Research Ethics Committee. Written informed consent was obtained from all adult participants. Moreover, written informed consent was obtained from a parent or legal guardian for participants aged <18 years.

### Data Collection

Eligible patients were contacted to participate in this study: 220 consented to participate as the *Shigella*-infected group, and 399 matched individuals consented to enroll as controls. Written informed consent was obtained from all adult participants. Moreover, written informed consent was obtained from a parent or legal guardian for participants aged <18 years. Demographic characteristics, including age, sex, employment status, education level, number of household members, and clinical characteristics during hospitalization (including nausea, vomiting, abdominal cramps, fever, duration and frequency of diarrhea, and hospitalization duration) were collected from the medical records in 2014. All participants were evaluated by the Rome-III questionnaire [23] for IBS and functional gastrointestinal diseases. The assessment of IBS conditions was conducted via a telephone interview instead of a face-to-face interview due to the coronavirus disease 2019 (COVID-19) pandemic. Data collection and interviews were carried out by 1 trained healthcare personnel. The incidence of IBS was compared between patients with and without a history of *Shigella* gastroenteritis in the 2014 *Shigella* outbreak, and risk factors were assessed in *Shigella*-infected participants.

### Statistical Analysis

The collected data were analyzed using IBM SPSS Statistics version 22 (IBM Corporation, Armonk, New York) and XLSTAT version 2022 (Addinsoft, New York, New York) software. Quantitative data were shown as mean and standard deviation (SD), and qualitative data were presented as frequency (percentages). For inferential analysis, odds ratios (ORs) and 95% confidence intervals (CIs) were utilized. The data were analyzed in 2 steps using univariate and multivariate logistic regression (forward stepwise). A *P* value of <.05 was considered to be significant.

## RESULTS

In this study, 619 participants were enrolled, of which 220 people (35.5%) were in the *Shigella*-infected group. The mean age of the participants in the control group was 41.03 (SD, 16.6) years, and in the *Shigella*-infected group was 42.26 (SD, 16.31) years. Women comprised 287 (65.3%) in the control group and 153 (69.8%) in the shigellosis group (Table 1).

**Table 1.** Characteristics of Patients and Controls

Variable	*Shigella*-Infected Group (n = 220)	Control Group (n = 399)	*P* Value
Age, y, mean (SD)	41.0 (16.6)	42.26 (16.3)	.361^a^
Sex (female), No. (%)	153 (69.8)	287 (65.3)	.531^b^
Education level, No. (%)			
No formal education	16 (7.2)	18 (4.5)	.317^b^
Primary/secondary education	67 (30.4)	118 (29.6)	
University	137 (62.3)	263 (65.9)	
Employment status (employed), No. (%)	185 (84.0)	312 (78.2)	.234^b^
Length of hospitalization, d, mean (SD)	5.2 (2.9)	4.8 (3.1)	.129^a^

Abbreviation: SD, standard deviation.

^a^Independent *t* test.

^b^χ^2^ test.

The 5-year incidence of IBS was 31.8% and 5.7% in the *Shigella*-infected and the control groups, respectively, which was a significant difference (*P* = .001). In the multivariate logistic regression model, adjustments were made for the possible confounders of age, sex, number of household members, employment status, and education level to investigate the association of shigellosis and IBS. This model showed that shigellosis was associated with IBS (OR, 17.18 [95% CI, 9.37–31.48]; *P*= .001).

Univariate logistic regression analysis was utilized to investigate the association of 12 theoretically predictive variables of IBS and its frequency (Table 2). Univariate analysis indicated that age (OR, 0.98 [95% CI, .97–.99]; *P* = .039), education level (OR, 3.44 [95% CI, 2.41–4.92]; *P* = .001), abdominal cramps (OR, 0.34 [95% CI, .18–.64]; *P* = .001), and frequency of diarrhea (OR, 1.50 [95% CI, 1.16–1.95]; *P* = .002) are significantly associated with IBS.

**Table 2.** Univariate Logistic Regression Analysis for Irritable Bowel Syndrome Risk Factors

Factor	Odds Ratio	(95% CI)	*P* Value
Age	0.98	(.97–.99)	.039
Male sex	1.11	…	.625
Education level	3.44	(4.41–4.92)	.001
Employment status	1.03	…	.521
No. of household members	1.11	…	.692
Abdominal cramps	0.34	(.18–.64)	.001
Nausea	1.77	…	.085
Vomiting	1.05	…	.865
Fever	0.77	…	.409
Frequency of diarrhea (per day)	1.50	(1.16–1.95)	.002
Diarrhea duration (days)	1.14	…	.141
Hospitalization duration (days)	1.36	…	.149

Abbreviation: CI, confidence interval.

A forward stepwise logistic regression model was utilized based on the univariate model results in the next step. In the final model, 3 variables of diarrhea duration (OR, 1.69 [95% CI, 1.17–2.44]; *P* = .005), education level (OR, 4.15 [95% CI, 1.47–11.73]; *P* = .007), and abdominal cramps (OR, 0.27 [95% CI, .77–.95]; *P* = .043) were assessed as IBS risk factors (Table 3). The model classified the variables with approximately 83% accuracy and explained 29% of the variation of IBS.

**Table 3.** Forward Stepwise Multivariate Logistic Regression Analysis for Irritable Bowel Syndrome Risk Factors

Factor	Odds Ratio	(95% CI)	*P* Value
Education level	4.15	(1.47–11.73)	.007
Abdominal cramps	0.27	(.77–.95)	.043
Diarrhea duration (days)	1.69	(1.17–2.44)	.005

**Figure 1.**
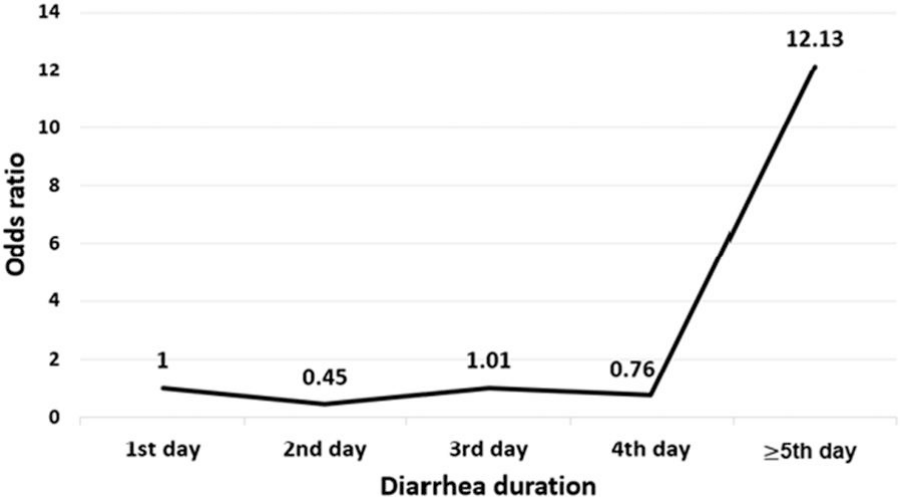
Risk of postinfectious irritable bowel syndrome based on days of diarrhea duration by univariate logistic regression.

After calculating the OR related to each day of prolonged diarrhea, it was shown that the risk of developing IBS will increase after the fifth day of diarrhea. Thus, in the first 4 days of diarrhea, there was no association between the 2 variables; however, after the fifth day, a 12-fold increase was observed in the risk of PI-IBS (Figure 1).

The statistical power was calculated as 1 for diarrhea for >4 days, 1 for higher education, and 1 for abdominal cramps, with 0.92, 0.80, and 0.78 probability, respectively.

## DISCUSSION

Our results indicated that the incidence of IBS 5 years after the *Shigella* outbreak was significantly higher among infected patients than in the control group. The odds of developing IBS in patients with shigellosis were approximately 17–31 times higher than in uninfected patients. Two statistical analysis methods were employed to investigate the association of potential predictive variables of PI-IBS. The univariate regression analysis indicated older age and the presence of abdominal cramps as protective factors, while education level and the frequency of diarrhea were identified as risk factors. The multivariate analysis showed that individuals with higher education levels and prolonged diarrhea during infection were likelier to develop PI-IBS, while those with abdominal pain were less likely. Our study also found that having diarrhea for >4 days was a significant risk factor for developing PI-IBS.

In a retrospective study by Iacob et al, which included 873 patients with positive stool cultures for gastroenteritis and a control group of 200 individuals, participants were evaluated using the Rome-III questionnaire 6 months after infection [16]. Their study found that the odds of developing IBS in patients with acute gastroenteritis were 4.1 times higher than in the control group, consistent with the findings of our study. Interestingly, in this study, female sex was associated with a higher risk of developing PI-IBS (relative risk, 4.4), in contrast to our results. This study utilized relative risk to assess the relationship between sex and PI-IBS. However, using ORs is recommended in retrospective studies to investigate the association between predictor and independent variables [24]. Furthermore, this study relied on univariate analysis, which does not consider the effect of confounding factors, potentially leading to errors in the results. A more robust approach would have been to use multivariate analysis, as we did in our study.

In a cohort study by Youn et al involving 124 patients with shigellosis, it was observed that these patients exhibited an 11.9-fold higher probability of developing IBS within the first year and a 3.9-fold increased risk of IBS in the third year following the infection [21]. However, no significant differences were found in the odds of developing IBS between infected and uninfected individuals during the fifth and eighth years postinfection. This study identified several independent risk factors associated with IBS, including a younger age, a history of functional gastrointestinal disorders, and the duration of diarrhea at the time of shigellosis [21]. Our findings are consistent with Youn and colleagues’ study in identifying a prolonged duration of diarrhea as a risk factor for PI-IBS. However, our study delved further into this association, revealing that the risk of IBS increased after the fifth day of diarrhea. Notably, our study employed an exclusion criterion for individuals with a history of functional gastrointestinal disorders, an identified risk factor in the previously mentioned study. Conversely, fever and vomiting were not found to have any significant association with PI-IBS in both studies. Education level and abdominal cramps were not investigated in the mentioned study.

Ji et al conducted a study involving 101 hospitalized patients with *Shigella* gastroenteritis and 102 participants in a control group. They assessed participants 3, 6, and 12 months following hospitalization [13]. The Rome-I questionnaire was utilized in this study to diagnose IBS. The findings revealed a significantly 2.5 times higher incidence of PI-IBS in patients with *Shigella* infection compared to the control group. Furthermore, their multivariate analysis identified the duration of diarrhea as a risk factor for PI-IBS (with a relative risk of 5.4 for prolonged diarrhea lasting >5 days compared to diarrhea lasting >2 days), which aligns with our findings. Similar to our study, age, sex, and vomiting were not found to be associated with PI-IBS. Notably, their study did not evaluate the impact of education level and abdominal cramps on PI-IBS.

Jung et al conducted a 5-year cohort study involving 119 *Shigella*-infected patients and a control group to investigate the incidence and related factors of PI-IBS. Age, sex, smoking, alcohol consumption, fever, vomiting, and duration of diarrhea during the infection did not significantly correlate with PI-IBS [25]. Notably, the results of Jung and colleagues’ study differed from ours concerning the relationship between the duration of diarrhea and PI-IBS, possibly due to the smaller sample size in their study.

Koh et al conducted a prospective study on 65 patients suffering from bacterial gastroenteritis. Of these patients, 33.8% had shigellosis. At 3 and 6 months after infection, the incidence of IBS in patients with gastroenteritis was similar to that of the general Western population [26]. Multivariate analysis identified prolonged diarrhea lasting >7 days as a risk factor for PI-IBS. Moreover, the study found no significant difference in abdominal pain and tenesmus during the infection between patients with and without PI-IBS.

Another study investigating 295 patients with bacillary dysentery, of which 71.4% were *Shigella* cases, showed a significantly higher incidence of PI-IBS in the dysentery group compared to the controls, with age and sex having no significant impact on the development of PI-IBS [27]. Furthermore, this study revealed prolonged diarrhea for >7 days associated with a 2.5-fold increase in the risk of functional gastrointestinal disorders.

In the systematic review and meta-analysis, several factors were identified as risk factors for PI-IBS, including depression (OR, 1.5), anxiety (OR, 1.9), female sex (OR, 2.2), abdominal pain during infection (OR, 3.2), antibiotic use during infection (OR, 1.7), bloody stool during infection (OR, 1.8), and prolonged diarrhea lasting >7 days during infection (OR, 2.6) [15]. In contrast, in our study, female sex and bloody stool were not identified as risk factors, and abdominal pain exhibited an inverse association with PI-IBS.

Upon comparison with previous studies, our study shows a higher risk of IBS following shigellosis. Notably, these previous studies frequently involved outpatients and hospital employees, whose disease severity was generally lower than that of the hospitalized patients in our investigation. Furthermore, we lack data regarding the preexisting IBS status of the patients examined before the *Shigella* outbreak, and their assessments were not standardized. Instead, we relied on inquiries about digestive symptoms before the onset of shigellosis. As a result, more *Shigella*-infected patients may have been exposed to the disease before the outbreak. Additionally, it is worth considering that the Iranian population is under considerable stress due to economic concerns, a high cost of living, and worries about employment and family prospects; a significant portion of the population in previous psychological studies exhibited symptoms of depression and anxiety. Moreover, a substantial proportion of these individuals experience severe stress. Hence, these factors may render patients more susceptible to developing IBS following a *Shigella* infection than prior cases [28, 29].

Our study revealed a direct association between education level and the risk of developing PI-IBS. Previous studies investigating the relationship between IBS and education level have shown conflicting results. Some studies have indicated a positive association between education level and IBS, while others have identified an inverse association, particularly in individuals with high incomes [30–37]. These inconsistent findings may be attributed to variations in how education levels are categorized and the use of different diagnostic criteria across studies. Regarding the direct association between IBS and education level, it is essential to note that anxiety, a significant risk factor for IBS, can be directly influenced by educational level [38]. Additionally, individuals with higher education levels may be more likely to hold positions that require greater expertise, potentially increasing occupational stress [39]. Therefore, higher education in our patients may contribute to stress, indirectly increasing the risk of IBS. While we did not assess patients’ income levels, studies suggest a possible link between wealth and higher risk of IBS in developing countries. Participants with higher education levels in our study likely had better financial standing [30]. This finding could also be influenced by recruitment bias among the population willing to participate in the study.

To elucidate the counterintuitive correlation between IBS and abdominal cramps, as identified in our study, it is essential to note that the retrospective nature of the study presents a challenge in relying solely on nonstandardized clinical assessments. This challenge arises due to the significant variability in the precise descriptions of gastrointestinal symptoms across different medical observations, including factors such as the exact number of stools per day, the duration of diarrhea, and the specific explanation of abdominal pain. It is crucial to emphasize that physicians specifically inquired about abdominal cramping only at the initiation of hospitalization and subsequently recorded it in the hospital records. As a result, this variable has not undergone a uniform evaluation with standardized questioning throughout the patient's hospital stay. Furthermore, we hypothesized that abdominal cramps, which tend to improve with defecation, may be less prevalent with more frequent diarrhea, a potential indicator of a more severe *Shigella* infection [7]. Additionally, it is worth considering that certain medications administered to alleviate gastroenteritis symptoms, such as bismuth subsalicylate, can exert an anti-inflammatory effect on gastroenteritis and influence the severity of the enteric illness, leading to a reduced incidence of PI-IBS [40, 41]. Notably, the onset of abdominal cramps might have occurred before hospital admission, prompting earlier hospitalization and more intensive care. Consequently, accelerated hospitalization and more intensive treatments could have led to more effective control of inflammation and infection, resulting in a lower incidence of PI-IBS in patients with abdominal cramps.

Our study had some limitations. Utilizing the most recent version of the Rome criteria for evaluating patients with IBS would have been advisable. However, this version was employed since the latest available translated version in Persian, which has been assessed for its validity and reliability, is Rome-III. The main limitation was the study's retrospective nature, which caused some methodological flaws. For instance, evaluating stress factors as a potential risk factor for IBS was impossible. Furthermore, patients were asked about having functional gastrointestinal disorders and symptoms before the outbreak, which can lead to recall bias. IBS was investigated in the studied patients using a standard questionnaire. Ideally, procedures like colonoscopy can help rule out other etiologies that may lead to gastrointestinal symptoms. Also, the lack of proper follow-up of patients did not allow us to examine the course of the disease and its prognosis. Thus, the patients who had symptoms of IBS during these 5 years and had spontaneous remission were considered non–PI-IBS patients. Hence, this study does not provide detailed information on the incidence and prevalence of all PI-IBS during all these years. This study was conducted only on hospitalized patients with more severe symptoms than outpatients, so valuable information on the incidence of PI-IBS in outpatients was missed. While investigating the factors associated with PI-IBS, it would have been preferable to calculate the association between each day of prolonged diarrhea after the fifth day and the incidence of PI-IBS. Such findings could have been represented in Figure 1. However, given that most of the patients experienced diarrhea for up to 5 days, and the number of cases with diarrhea persisting beyond this period was limited, we were unable to achieve this goal. For instance, we could not determine the association between PI-IBS and diarrhea that persisted for 6, 7, or 8 days. Furthermore, in this study, some variables theoretically related to PI-IBS, such as bloody diarrhea, depression, history of appendicitis and previous hysterectomy, and characteristics of the childhood homes, were not evaluated in our study [42]. The mentioned variables can confound the results. Ideally, it would be beneficial to include an investigation of major comorbidities, such as diabetes and immunosuppression, to enhance the study results’ precision and accuracy. Our study did not evaluate the treatment measures for patients during hospitalization. Another variable that remains unexplored in this study is the volume of fluid therapy during hospitalization, which can influence certain IBS-related factors, such as fever. Furthermore, conducting a multicenter study at the national level was unfeasible due to the *Shigella* outbreak being localized to a single city.

Despite these limitations, it is essential to highlight the clinical significance of this study. It can aid in predicting and identifying patients at risk for PI-IBS who may require more intensive follow-up. Studies focusing on the incidence of PI-IBS following *Shigella* infection have been scarce, primarily concentrated in South Korea, with some of these studies neglecting to investigate potential predictive factors. Therefore, our study contributes to a more comprehensive understanding of the risk factors associated with PI-IBS. Unlike similar studies that have examined infected patients with various pathogens, diverse clinical symptoms, and involvement of different segments of the digestive system, our study concentrated on patients infected with a specific pathogen and a defined spectrum of clinical symptoms. Our study's high statistical power enables accurate conclusions. It is recommended to conduct further multicenter studies that consider all potential variables associated with PI-IBS to attain more precise and generalizable results.

In conclusion, future studies could explore the potential impact of *Shigella* spp on the patient's microbiome. Analyzing the gastrointestinal flora after infection in subsequent studies could provide deeper insights into the pathogenesis of PI-IBS and inform the development of novel treatment strategies for this condition.

## Notes

***Acknowledgments.*** We gratefully acknowledge all patients who participated in this study and thank all of the health professionals who helped with the process of this study and data collection.

***Author contributions.*** M. S., P. A., and H. M. designed the study. H. M., N. G., H. R., and A. C. screened the patients and collected the study data. N. G. and A. C. analyzed the clinical data and wrote the manuscript. All authors read the manuscript and approved the final draft that was submitted.

***Data availability.*** Due to confidentiality agreements, supporting data can only be made available to bona fide researchers subject to a nondisclosure agreement. Details of the data and how to request access can be obtained by contacting the corresponding author.
